# The infimum values of two probability functions for the Gamma distribution

**DOI:** 10.1186/s13660-024-03081-w

**Published:** 2024-01-17

**Authors:** Ping Sun, Ze-Chun Hu, Wei Sun

**Affiliations:** 1 Business School, Chengdu University, Chengdu, 610106 China; 2 College of Mathematics, Sichuan University, Chengdu, 610065 China; 3 Department of Mathematics and Statistics, Concordia University, Montreal, H3G 1M8 Canada

**Keywords:** 60E15, 62G32, 90C15, Gamma distribution, Infinitely divisible distribution, Infimum value, Probability inequality

## Abstract

Let *α*, *β* be positive real numbers and let $X_{\alpha ,\beta}$ be a Gamma random variable with shape parameter *α* and scale parameter *β*. We study infimum values of the function $(\alpha ,\beta )\mapsto P\{X_{\alpha ,\beta}\le \kappa E[X_{\alpha ,\beta}] \}$ for any fixed $\kappa >0$ and the function $(\alpha ,\beta )\mapsto P\{|X_{\alpha ,\beta}-E[X_{\alpha ,\beta}]| \le \sqrt{\operatorname{Var}(X_{\alpha ,\beta})}\}$. We show that $\inf_{\alpha ,\beta}P\{X_{\alpha ,\beta}\le E[X_{\alpha ,\beta}]\}= \frac{1}{2}$ and $\inf_{\alpha ,\beta}P\{|X_{\alpha ,\beta}-E[X_{\alpha ,\beta}]|\le \sqrt{\operatorname{Var}(X_{\alpha ,\beta})}\}=P\{|Z|\le 1\}\approx 0.6827$, where *Z* is a standard normal random variable.

## Introduction

Special probability distributions play a fundamental role in probability theory, statistics, optimization, and different research fields of science, including physics, chemistry, bioscience, economy, and management science. Although they have been studied for a long time, our understanding of them is far from complete. This paper is motivated by Chvátal’s conjecture for the binomial distribution and Tomaszewaki’s conjecture for the Rademacher sequence, both of which were completely solved very recently.

Let $B(n,p)$ denote a binomial random variable with parameters *n* and *p*. Janson [5] introduced the following conjecture suggested by Vašk Chvátal.

### Conjecture 1

(Chvátal)

*For any fixed* $n\geq 2$, *as* *m* *ranges over* $\{0,\ldots ,n\}$, *the probability* $q_{m}:=P\{B(n,\frac{m}{n})\leq m\}$ *is the smallest when* *m* *is the integer closest to* $\frac{2n}{3}$.

Chvátal’s conjecture has interesting applications in machine learning. Janson [5] proved Chvátal’s conjecture for sufficiently large *n*. Barabesi et al. [1] and Sun [12] showed that Chvátal’s conjecture is true for any $n\geq 2$.

The second motivation of this paper is the following problem, attributed to Boguslav Tomaszewski.

### Conjecture 2

(Tomaszewski)

*Let* $X=\sum_{i=1}^{n}a_{i}X_{i}$, *where* $\sum_{i=1}^{n} a_{i}^{2}=1$ *and* $\{X_{i}, i\ge 1\}$ *is a sequence of independent* $\{-1,1\}$-*valued symmetric random variables*. *Then* $P\{|X|\leq 1\}\geq 1/2$.

Tomaszewski’s conjecture has many applications in probability theory, geometric analysis, and computer science. Recently, Keller and Klein [6] completely solved Tomaszewski’s conjecture. We refer the reader to Keller and Klein [6] for the details and to Dvorak and Klein [3] and Hu et al. [4] for some related problems.

In this paper, we will focus on the Gamma distribution. It is well known that the Gamma distribution, including the exponential distribution and the $\chi ^{2}$-distribution as two important special cases, is one of the most basic probability distributions (cf. [7, Chap. 16]). It is frequently applied to describe the time between independent events that occur at a constant average rate. The Gamma distribution has many significant applications. For example, it has been used to model the size of insurance claims, rainfall, failure times of repairable systems, load levels for telecommunication services, and the distribution of asset prices.

Motivated by Chvátal’s conjecture, Li et al. [9] initiated the study of the infimum value problem for special probability distributions. Let $\{Y_{\lambda},\lambda >0\}$ be a family of random variables with the same distribution *F* but different parameters *λ*. Define

$$ r(\lambda ):=P\bigl\{ Y_{\lambda}\le E[Y_{\lambda}]\bigr\} . $$

Li et al. [9] discussed the infimum value of the function *r* and gave a complete answer if *F* is the Poisson distribution or the geometric distribution. Further, Li et al. [8] considered the infimum value problem for the Weibull and Pareto distributions.

In the first part of this paper, we will consider the following more general infimum value problem for the Gamma distribution. Let $\alpha ,\beta ,\kappa >0$ and let $X_{\alpha ,\beta}$ be a Gamma random variable with shape parameter *α* and scale parameter *β*. Define

1.1 $$ g_{\kappa}(\alpha ,\beta ):=P\bigl\{ X_{\alpha ,\beta} \le \kappa E[X_{\alpha ,\beta}] \bigr\} . $$

**Figure 1:**
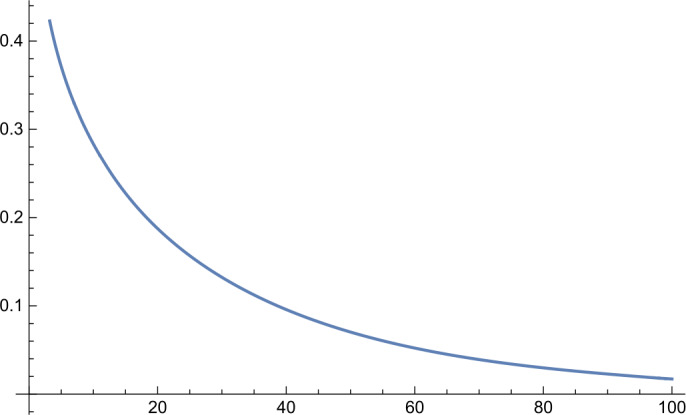
Function $h_{\kappa}(\alpha )$ for $\kappa =0.8$

**Figure 2:**
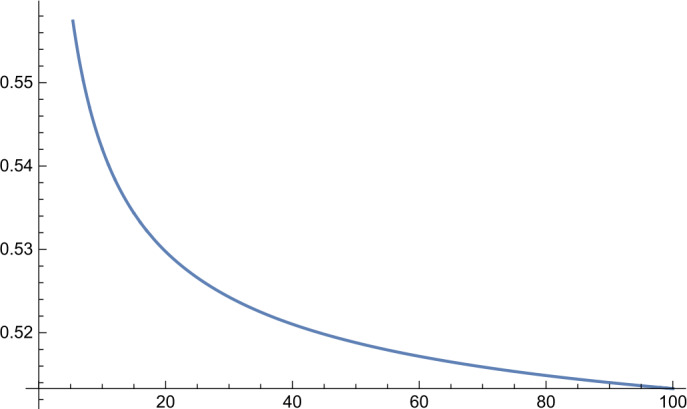
Function $h_{\kappa}(\alpha )$ for $\kappa =1$

**Figure 3:**
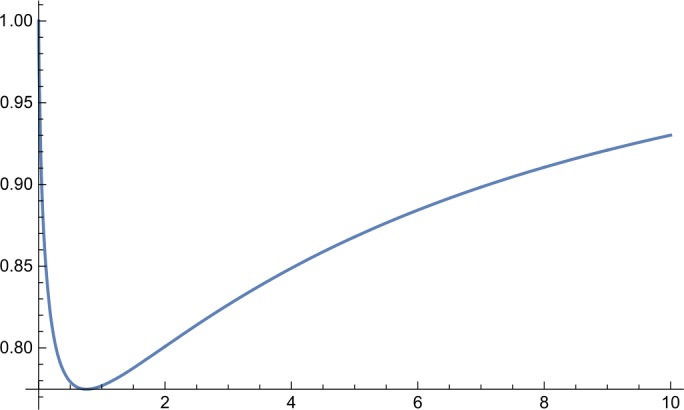
Function $h_{\kappa}(\alpha )$ for $\kappa =1.5$

**Figure 4:**
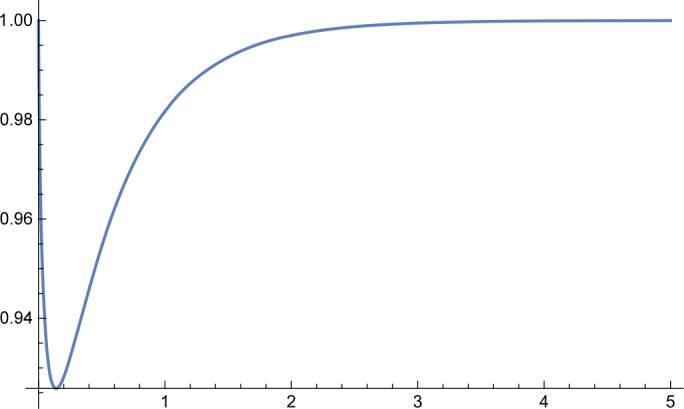
Function $h_{\kappa}(\alpha )$ for $\kappa =4$

For fixed *κ*, what is the infimum value of the function $g_{\kappa}(\alpha ,\beta )$? In Sect. 2, we will give a complete answer to this question. Interestingly, we discover an unnoticed phase transition phenomenon (cf. Figs. 1–4 and Remark 2.2) and obtain the following result.

### Theorem 1.1

*Let* *α*, *β* *be arbitrary positive real numbers and let* $X_{\alpha ,\beta}$ *be a Gamma random variable with shape parameter* *α* *and scale parameter* *β*. *Then*

1.2 $$ P\bigl\{ X_{\alpha ,\beta}\le E[X_{\alpha ,\beta}]\bigr\} > \frac{1}{2} $$

*and*

$$ \inf_{\alpha ,\beta}P\bigl\{ X_{\alpha ,\beta}\le E[X_{\alpha ,\beta}] \bigr\} = \frac{1}{2}. $$

In the second part of this paper, we will prove a more interesting and deeper result.

### Theorem 1.2

*Let* *α*, *β* *be arbitrary positive real numbers*, *let* $X_{\alpha ,\beta}$ *be a Gamma random variable with shape parameter* *α* *and scale parameter* *β*, *and let* *Z* *be a standard normal random variable*. *Then*

1.3 $$ P \bigl\{ \bigl\vert X_{\alpha ,\beta}-E[X_{\alpha ,\beta}] \bigr\vert \le \sqrt{\operatorname{Var}(X_{\alpha ,\beta})} \bigr\} >P\bigl\{ \vert Z \vert \le 1\bigr\} \approx 0.6827 $$

*and*

$$ \inf_{\alpha ,\beta}P \bigl\{ \bigl\vert X_{\alpha ,\beta}-E[X_{\alpha ,\beta}] \bigr\vert \le \sqrt{\operatorname{Var}(X_{\alpha ,\beta})} \bigr\} =P\bigl\{ \vert Z \vert \le 1\bigr\} . $$

We would like to point out that if $0<\kappa \neq1$, then the following more general inequality might not hold for all Gamma random variables:

$$ P \bigl\{ \bigl\vert X_{\alpha ,\beta}-E[X_{\alpha ,\beta}] \bigr\vert \le \kappa \sqrt{ \operatorname{Var}(X_{\alpha ,\beta})} \bigr\} >P\bigl\{ \vert Z \vert \le \kappa \bigr\} . $$

For example, we have

$$\begin{aligned} 0.3834005&=P \bigl\{ \bigl\vert X_{1,1}-E[X_{1,1}] \bigr\vert \le 0.5\sqrt{\operatorname{Var}(X_{1,1})} \bigr\} \\ & > 0.3829249=P\bigl\{ \vert Z \vert \le 0.5\bigr\} \\ & > 0.3819693=P \bigl\{ \bigl\vert X_{2,1}-E[X_{2,1}] \bigr\vert \le 0.5\sqrt{\operatorname{Var}(X_{2,1})} \bigr\} \end{aligned}$$

and

$$\begin{aligned} 0.9502129&=P \bigl\{ \bigl\vert X_{1,1}-E[X_{1,1}] \bigr\vert \le 2\sqrt{\operatorname{Var}(X_{1,1})} \bigr\} \\ & < 0.9544997=P\bigl\{ \vert Z \vert \le 2\bigr\} \\ & < 0.9585112=P \bigl\{ \bigl\vert X_{10,1}-E[X_{10,1}] \bigr\vert \le 2\sqrt{\operatorname{Var}(X_{10,1})} \bigr\} . \end{aligned}$$

Note that the Gamma distribution is unimodal (cf. [10, Example 23.3]). The Vysochanskiĭ–Petunin inequality for unimodal distributions (cf. [2, Theorem 1.12] and [15]) tells us that

1.4 $$ P \bigl\{ \bigl\vert X_{\alpha ,\beta}-E[X_{\alpha ,\beta}] \bigr\vert \le \kappa \sqrt{ \operatorname{Var}(X_{\alpha ,\beta})} \bigr\} \ge 1-\max \biggl\{ \frac{4}{3\kappa ^{2}}-\frac{1}{3},\frac{4}{9\kappa ^{2}} \biggr\} . $$

The estimate (1.4) is non-trivial if $\kappa >1$; however, it yields a lower bound of 0 for the probabilities $P\{|X_{\alpha ,\beta}-E[X_{\alpha ,\beta}]|\le \sqrt{\operatorname{Var}(X_{\alpha ,\beta})} \}$, $\alpha ,\beta >0$.

The remainder of this paper is organized as follows. In Sect. 2, we investigate the infimum value problem for the function $g_{\kappa}(\alpha ,\beta )$ and give the proof of Theorem 1.1. In Sects. 3 and 4, we discuss the variation comparison between the Gamma distribution and the normal distribution and give the proof of Theorem 1.2. In Sect. 5, we make concluding remarks. The software *Mathematica* has been used in Sect. 4. Some *Mathematica* calculations are given in the Appendix.

## The infimum value problem for the function $g_{\kappa}(\alpha ,\beta )$

Let $\alpha ,\beta >0$ and let $X_{\alpha ,\beta}$ be a Gamma random variable with probability density function

$$ f_{\alpha ,\beta}(x)= \frac{x^{\alpha -1}e^{-x/\beta}}{\Gamma (\alpha )\beta ^{\alpha}},\quad x>0. $$

For $\kappa >0$, we consider the infimum value of the probability function $g_{\kappa}(\alpha ,\beta )$ defined by (1.1).

We have

$$\begin{aligned} g_{\kappa}(\alpha ,\beta )= \int _{0}^{\kappa \alpha \beta} \frac{x^{\alpha -1}e^{-x/\beta}}{\Gamma (\alpha )\beta ^{\alpha}}\,dx = \int _{0}^{\kappa \alpha}\frac{y^{\alpha -1}e^{-y}}{\Gamma (\alpha )}\,dy =g_{\kappa}( \alpha ,1). \end{aligned}$$

Then we may assume without loss of generality that $\beta =1$ and focus on the infimum value of the following function:

$$ h_{\kappa}(\alpha ):=g_{\kappa}(\alpha ,1)= \int _{0}^{\kappa \alpha} \frac{y^{\alpha -1}e^{-y}}{\Gamma (\alpha )}\,dy ,\quad \alpha >0. $$

By Euler’s reflection formula

$$ \Gamma (1-\alpha )\Gamma (\alpha )=\frac{\pi}{\sin (\pi \alpha )},\quad \alpha \in (0,1), $$

we get

2.1 $$\begin{aligned} \liminf_{\alpha \downarrow 0}h_{\kappa}(\alpha ) & = \liminf_{\alpha \downarrow 0} \int _{0}^{\kappa \alpha}\alpha y^{\alpha -1}e^{-y}\,dy \\ & \ge \liminf_{\alpha \downarrow 0} \biggl[\alpha e^{-\kappa \alpha} \int _{0}^{\kappa \alpha} y^{\alpha -1}\,dy \biggr] \\ & = \liminf_{\alpha \downarrow 0}(\kappa \alpha )^{\alpha} \\ & = \liminf_{\alpha \downarrow 0}e^{\alpha \ln (\kappa \alpha )} \\ & = 1. \end{aligned}$$

It follows that for any $\kappa >0$, $\sup_{\alpha >0}h_{\kappa}(\alpha )=1$.

### Case $\kappa \le 1$

In this subsection, we assume that $\kappa \le 1$. We will show that $h_{\kappa}(\alpha +1)< h_{\kappa}(\alpha )$ for any $\alpha >0$. In fact, we have

2.2 $$\begin{aligned} h_{\kappa}(\alpha +1)< h_{\kappa}(\alpha ) & \quad \Leftrightarrow\quad \Gamma ( \alpha +1)\bigl[h_{\kappa}(\alpha +1)-h_{\kappa}(\alpha )\bigr]< 0 \\ & \quad \Leftrightarrow\quad \int _{0}^{\kappa (\alpha +1)}y^{\alpha}e^{-y}\,dy - \alpha \int _{0}^{\kappa \alpha}y^{\alpha -1}e^{-y}\,dy < 0 \\ & \quad \Leftrightarrow\quad -\bigl[\kappa (\alpha +1)\bigr]^{\alpha}e^{-\kappa (\alpha +1)}+ \alpha \int _{\kappa \alpha}^{\kappa (\alpha +1)}y^{\alpha -1}e^{-y}\,dy < 0 \\ & \quad \Leftrightarrow\quad \alpha \int _{\kappa \alpha}^{\kappa (\alpha +1)}y^{\alpha -1}e^{\kappa (\alpha +1)-y}\,dy < \bigl[ \kappa (\alpha +1)\bigr]^{\alpha} \\ & \quad \Leftrightarrow\quad \alpha \int _{0}^{\kappa}\bigl[\kappa (\alpha +1)-w \bigr]^{\alpha -1}e^{w}\,dw < \bigl[ \kappa (\alpha +1) \bigr]^{\alpha} \\ & \quad \Leftrightarrow\quad - \int _{0}^{\kappa}e^{w}d\bigl[\kappa ( \alpha +1)-w\bigr]^{\alpha}< \bigl[ \kappa (\alpha +1)\bigr]^{\alpha} \\ & \quad \Leftrightarrow\quad \int _{0}^{\kappa}\bigl[\kappa (\alpha +1)-w \bigr]^{\alpha}e^{w}\,dw < ( \kappa \alpha )^{\alpha}e^{\kappa} \\ & \quad \Leftrightarrow\quad \int _{0}^{\kappa} \biggl(1+ \frac{\kappa -w}{\kappa \alpha} \biggr)^{\alpha}e^{w-\kappa}\,dw < 1 \\ & \quad \Leftrightarrow\quad \int _{0}^{\kappa} \biggl(1+\frac{z}{\kappa \alpha} \biggr)^{\alpha}e^{-z}\,dz < 1 \\ & \quad \Leftrightarrow\quad \int _{0}^{1}\kappa \biggl(1+ \frac{w}{\alpha} \biggr)^{\alpha}e^{-\kappa w}\,dw < 1. \end{aligned}$$

Note that $(1+\frac{1}{x})^{x}$ is strictly increasing with respect to $x>0$ and the limit equals *e*. Then we have

$$ \biggl(1+\frac{w}{\alpha} \biggr)^{\alpha}= \biggl\{ \biggl(1+ \frac{w}{\alpha} \biggr)^{\frac{\alpha}{w}} \biggr\} ^{w}< e^{w}. $$

Thus,

2.3 $$\begin{aligned} \int _{0}^{1}\kappa \biggl(1+ \frac{w}{\alpha} \biggr)^{\alpha}e^{-\kappa w}\,dw < \int _{0}^{1}e^{w}\cdot \kappa e^{-\kappa w}\,dw \le \int _{0}^{1}e^{w} \cdot e^{- w}\,dw =1. \end{aligned}$$

Hence, by (2.2) and (2.3), we obtain that $\{h_{\kappa}(\alpha +n)\}_{n\in \mathbb{N}}$ is a strictly decreasing sequence.

Let $Y_{0},Y_{1},Y_{2},\dots $ be independent random variables such that $Y_{0}\sim \operatorname{Gamma} (\alpha ,1)$ and $Y_{i}\sim \operatorname{Gamma} (1,1)$, $i\ge 1$. Then, for $\kappa \in (0,1)$, by the strong law of large numbers, we get

$$\begin{aligned} \lim_{n\rightarrow \infty}h_{\kappa}(\alpha +n) & = \lim _{n\rightarrow \infty}P \bigl\{ Y_{0}+Y_{1}+\cdots +Y_{n}\le \kappa E[Y_{0}+Y_{1}+\cdots +Y_{n}]\bigr\} \\ & = \lim_{n\rightarrow \infty}P \biggl\{ \frac{Y_{0}}{\alpha +n}+ \frac{Y_{1}+\cdots +Y_{n}}{\alpha +n}\le \kappa \biggr\} \\ & = 0, \end{aligned}$$

and for $\kappa =1$, by the central limit theorem, we get

$$\begin{aligned} \lim_{n\rightarrow \infty}h_{\kappa}(\alpha +n) & = \lim _{n\rightarrow \infty}P \bigl\{ Y_{0}+Y_{1}+\cdots +Y_{n}\le E[Y_{0}+Y_{1}+\cdots +Y_{n}]\bigr\} \\ & = \lim_{n\rightarrow \infty}P\bigl\{ (Y_{0}-\alpha )+(Y_{1}-1)+\cdots +(Y_{n}-1) \le 0\bigr\} \\ & = \lim_{n\rightarrow \infty}P \biggl\{ \frac{Y_{0}-\alpha}{\sqrt{n}}+ \frac{(Y_{1}-1)+\cdots +(Y_{n}-1)}{\sqrt{n}}\le 0 \biggr\} \\ & = \frac{1}{2}. \end{aligned}$$

Therefore,

$$\begin{aligned} \inf_{\alpha >0}h_{\kappa}(\alpha )=\textstyle\begin{cases} 0, & \kappa \in (0,1), \\ \frac{1}{2}, & \kappa =1,\end{cases}\displaystyle \end{aligned}$$

which implies that inequality (1.2) holds. The proof of Theorem 1.1 is complete.

#### Remark 2.1

It is interesting to compare (1.2) with the well-known fact that the Gamma distribution is skewed to the right and its skewness is given by

$$ E \biggl[ \biggl( \frac{X_{\alpha ,\beta}-E[X_{\alpha ,\beta}]}{\sqrt{\operatorname{Var}(X_{\alpha ,\beta})}} \biggr)^{3} \biggr]= \frac{2}{\sqrt{\alpha}}. $$

### Case $\kappa > 1$

In this subsection, we assume that $\kappa > 1$. This case is especially interesting. By (1.2), for any $\alpha >0$, we have

2.4 $$\begin{aligned} h_{\kappa}(\alpha )=P\bigl\{ X_{\alpha ,1}\le \kappa E[X_{\alpha ,1}]\bigr\} >P\bigl\{ X_{\alpha ,1} \le E[X_{\alpha ,1}]\bigr\} >\frac{1}{2}. \end{aligned}$$

Let $Y_{1},Y_{2},\dots $ be independent $\operatorname{Gamma} (1,1)$ random variables. By the central limit theorem, we get

2.5 $$\begin{aligned} \liminf_{\alpha \rightarrow \infty}h_{\kappa}(\alpha ) & \ge \liminf_{\alpha \rightarrow \infty}P\bigl\{ Y_{1}+\cdots +Y_{[\alpha ]+1} \le \kappa [\alpha ]\bigr\} \\ & = \lim_{n\rightarrow \infty}P \biggl\{ \frac{(Y_{1}-1)+\cdots +(Y_{n+1}-1)}{\sqrt{n+1}}\le \frac{(\kappa -1)n-1}{\sqrt{n+1}} \biggr\} \\ & = 1. \end{aligned}$$

Hence, by (2.1), (2.4), and (2.5), we obtain

$$ \min_{\alpha >0}h_{\kappa}(\alpha )>\frac{1}{2}, \quad \forall \kappa >1. $$

Further, by virtue of *Mathematica*, we obtain the following numerical results:

$$ \min_{\alpha >0}h_{\kappa}(\alpha )=\textstyle\begin{cases} h_{\kappa}(33.4871)=0.545885, & \kappa =1.01, \\ h_{\kappa}(3.47146)=0.64021, & \kappa =1.1, \\ h_{\kappa}(1.78959)=0.691283, & \kappa =1.2, \\ h_{\kappa}(0.757559)=0.774739, & \kappa =1.5, \\ h_{\kappa}(0.396184)=0.841243, & \kappa =2, \\ h_{\kappa}(0.205464)=0.899108, & \kappa =3, \\ h_{\kappa}(0.13917)=0.925864, & \kappa =4.\end{cases} $$

Graphs of the function $h_{\kappa}(\alpha )$ for different values of *κ* are shown in Figs. 1–4.

#### Remark 2.2

The above analysis shows that there is an interesting phase transition phenomenon in the infimum value problem for the Gamma distribution. The critical point is $\kappa =1$ and the behaviors for the three cases $\kappa <1$, $\kappa =1$, and $\kappa >1$ are totally different.

## Variation comparison between the Gamma distribution and the normal distribution

The empirical rule tells us that in an approximately normal distribution, about 68% of the values fall within one standard deviation of the mean, about 95% of the values fall within two standard deviations of the mean, and almost all values fall within three standard deviations of the mean. A question worth thinking about is what we can say about other distributions. Theorem 1.2 compares the variation between the Gamma distribution and the normal distribution. In this section, we will prove Theorem 1.2.

Let $\alpha ,\beta >0$. Define

$$ s(\alpha ,\beta ):=P \bigl\{ \bigl\vert X_{\alpha ,\beta}-E[X_{\alpha ,\beta}] \bigr\vert \le \sqrt{\operatorname{Var}(X_{\alpha ,\beta})} \bigr\} . $$

We have

$$\begin{aligned} s(\alpha ,\beta )=P\bigl\{ \vert X_{\alpha ,\beta}-\alpha \beta \vert \le \sqrt{ \alpha}\beta \bigr\} =P \biggl\{ \biggl\vert \frac{X_{\alpha ,\beta}}{\beta}- \alpha \biggr\vert \le \sqrt{\alpha} \biggr\} =s(\alpha ,1). \end{aligned}$$

Then we may assume without loss of generality that $\beta =1$ and focus on the following function:

$$ t(\alpha ):=s(\alpha ,1)= \int _{\max \{0,\alpha -\alpha ^{\frac{1}{2}}\}}^{\alpha +\alpha ^{\frac{1}{2}}} \frac{y^{\alpha -1}e^{-y}}{\Gamma (\alpha )}\,dy ,\quad \alpha >0. $$

This section is devoted to proving the following result.

### Theorem 3.1

*For any* $\alpha >0$,

3.1 $$ t(\alpha +1)< t(\alpha ). $$

Note that by the central limit theorem, $\lim_{\alpha \rightarrow \infty}t(\alpha )=P\{|Z|\leq 1\}$, where *Z* is a standard normal random variable. Once (3.1) is proved, we conclude that

$$ s(\alpha ,\beta )=s(\alpha ,1)=t(\alpha )>P\bigl\{ \vert Z \vert \leq 1\bigr\} \approx 0.6827, \quad \forall \alpha ,\beta >0. $$

Thus, inequality (1.3) holds and hence the proof of Theorem 1.2 is complete.

### Proof of Theorem 3.1

First, we consider the case $0<\alpha \le 1$. We have

3.2 $$\begin{aligned} &t(\alpha +1)< t(\alpha ) \\ & \quad \Leftrightarrow\quad \Gamma (\alpha +1)\bigl[t(\alpha +1)-t(\alpha )\bigr]< 0 \\ & \quad \Leftrightarrow\quad \int _{\alpha +1-{(\alpha +1)}^{\frac{1}{2}}}^{\alpha +1+(\alpha +1)^{\frac{1}{2}}}y^{\alpha}e^{-y}\,dy - \alpha \int _{0}^{\alpha +\alpha ^{\frac{1}{2}}}y^{\alpha -1}e^{-y}\,dy < 0 \\ & \quad \Leftrightarrow\quad \int _{\alpha +1-{(\alpha +1)}^{\frac{1}{2}}}^{\alpha +1+(\alpha +1)^{\frac{1}{2}}}y^{\alpha}e^{-y}\,dy - \int _{0}^{\alpha +\alpha ^{\frac{1}{2}}}y^{\alpha}e^{-y}\,dy < \bigl(\alpha + \alpha ^{\frac{1}{2}}\bigr)^{\alpha}e^{-\alpha -\alpha ^{\frac{1}{2}}} \\ & \quad \Leftrightarrow\quad \int _{\alpha +\alpha ^{\frac{1}{2}}}^{\alpha +1+(\alpha +1)^{\frac{1}{2}}}y^{\alpha}e^{-y}\,dy < \int _{0}^{\alpha +1-{(\alpha +1)}^{\frac{1}{2}}}y^{\alpha}e^{-y}\,dy + \bigl( \alpha +\alpha ^{\frac{1}{2}}\bigr)^{\alpha}e^{-\alpha -\alpha ^{\frac{1}{2}}} \\ & \quad \Leftarrow\quad \int _{\alpha +\alpha ^{\frac{1}{2}}}^{\alpha +1+(\alpha +1)^{\frac{1}{2}}}y^{\alpha}e^{-y}\,dy < \bigl( \alpha +\alpha ^{\frac{1}{2}}\bigr)^{\alpha}e^{-\alpha -\alpha ^{\frac{1}{2}}} \\ & \quad \Leftrightarrow\quad \int _{0}^{1+(\alpha +1)^{\frac{1}{2}}-\alpha ^{\frac{1}{2}}} \biggl(1+\frac{w}{\alpha +\alpha ^{\frac{1}{2}}} \biggr)^{\alpha}e^{-w}\,dw < 1 \\ & \quad \Leftarrow\quad \int _{0}^{1+(\alpha +1)^{\frac{1}{2}}-\alpha ^{\frac{1}{2}}} \biggl(1+\frac{\alpha w}{\alpha +\alpha ^{\frac{1}{2}}} \biggr)e^{-w}\,dw < 1 \\ & \quad \Leftrightarrow\quad \frac{\alpha}{\alpha +\alpha ^{\frac{1}{2}}}- \frac{3\alpha +\alpha (\alpha +1)^{\frac{1}{2}}+(1-\alpha )\alpha ^{\frac{1}{2}}}{\alpha +\alpha ^{\frac{1}{2}}} \cdot e^{-1-(\alpha +1)^{\frac{1}{2}}+\alpha ^{\frac{1}{2}}}< 0 \\ & \quad \Leftrightarrow\quad e^{1+(\alpha +1)^{\frac{1}{2}}-\alpha ^{\frac{1}{2}}}< 3+( \alpha +1)^{\frac{1}{2}}+(1-\alpha )\alpha ^{-\frac{1}{2}} \\ & \quad \Leftrightarrow\quad e^{1+(\alpha +1)^{\frac{1}{2}}-\alpha ^{\frac{1}{2}}}-\bigl[1+( \alpha +1)^{\frac{1}{2}}- \alpha ^{\frac{1}{2}}\bigr]< 2+\alpha ^{-\frac{1}{2}}. \end{aligned}$$

Set

$$ w:=(\alpha +1)^{\frac{1}{2}}-\alpha ^{\frac{1}{2}}. $$

Then

$$ 0< w< 1\quad \text{and} \quad \alpha ^{-\frac{1}{2}}=\frac{2w}{1-w^{2}}. $$

Hence,

3.3 $$\begin{aligned} &2+\alpha ^{-\frac{1}{2}}- \bigl\{ e^{1+(\alpha +1)^{\frac{1}{2}}-\alpha ^{\frac{1}{2}}}- \bigl[1+(\alpha +1)^{\frac{1}{2}}-\alpha ^{\frac{1}{2}} \bigr] \bigr\} \\ &\quad = \frac{3+3w-3w^{2}-w^{3}}{1-w^{2}}-e^{1+w} \\ &\quad > \frac{3+3w-3w^{2}-w^{3}}{1-w^{2}}- \Biggl[\sum_{n=0}^{4} \frac{(1+w)^{n}}{n!}+ \frac{2^{5}}{5!} \Biggr] \\ &\quad = \frac{3 + 40 w - 153 w^{2} + 160 w^{3} + 145 w^{4} + 40 w^{5} + 5 w^{6}}{120(1-w^{2})} . \end{aligned}$$

Define

$$ I:=3 + 40 w - 153 w^{2} + 160 w^{3} + 145 w^{4} + 40 w^{5} + 5 w^{6}. $$

Set

$$\begin{aligned} w:=\frac{1}{1+q^{2}}. \end{aligned}$$

Then we have

$$\begin{aligned} \bigl(1 + q^{2}\bigr)^{6}I=240 + 416 q^{2} + 152 q^{4} + 8 q^{6} + 92 q^{8} + 58 q^{10} + 3 q^{12}>0, \end{aligned}$$

which together with (3.2) and (3.3) implies that (3.1) holds for the case $0<\alpha \le 1$.

Next we consider the case $\alpha >1$. We have

$$\begin{aligned} &t(\alpha +1)< t(\alpha ) \\ & \quad \Leftrightarrow\quad \Gamma (\alpha +1)\bigl[t(\alpha +1)-t(\alpha )\bigr]< 0 \\ & \quad \Leftrightarrow\quad \int _{\alpha +1-{(\alpha +1)}^{\frac{1}{2}}}^{\alpha +1+(\alpha +1)^{\frac{1}{2}}}y^{\alpha}e^{-y}\,dy - \alpha \int _{\alpha -\alpha ^{\frac{1}{2}}}^{\alpha +\alpha ^{\frac{1}{2}}}y^{\alpha -1}e^{-y}\,dy < 0 \\ & \quad \Leftrightarrow\quad \int _{\alpha +1-{(\alpha +1)}^{\frac{1}{2}}}^{\alpha +1+(\alpha +1)^{\frac{1}{2}}}y^{\alpha}e^{-y}\,dy - \int _{\alpha -\alpha ^{\frac{1}{2}}}^{\alpha +\alpha ^{\frac{1}{2}}}y^{\alpha}e^{-y}\,dy < \bigl( \alpha +\alpha ^{\frac{1}{2}}\bigr)^{\alpha}e^{-\alpha -\alpha ^{\frac{1}{2}}}- \bigl( \alpha -\alpha ^{\frac{1}{2}}\bigr)^{\alpha}e^{-\alpha +\alpha ^{\frac{1}{2}}} \\ & \quad \Leftrightarrow\quad \int _{\alpha +\alpha ^{\frac{1}{2}}}^{\alpha +1+(\alpha +1)^{\frac{1}{2}}}y^{\alpha}e^{-y}\,dy - \bigl( \alpha +\alpha ^{\frac{1}{2}}\bigr)^{\alpha}e^{-\alpha -\alpha ^{\frac{1}{2}}} \\ &\hphantom{ \quad \Leftrightarrow\quad}\quad < \int _{\alpha -\alpha ^{\frac{1}{2}}}^{\alpha +1-{(\alpha +1)}^{\frac{1}{2}}}y^{\alpha}e^{-y}\,dy - \bigl( \alpha -\alpha ^{\frac{1}{2}}\bigr)^{\alpha}e^{-\alpha +\alpha ^{\frac{1}{2}}} \\ & \quad \Leftrightarrow\quad \bigl(\alpha +\alpha ^{\frac{1}{2}} \bigr)^{\alpha}e^{-\alpha -\alpha ^{\frac{1}{2}}} \biggl[ \int _{0}^{1+(\alpha +1)^{\frac{1}{2}}-\alpha ^{\frac{1}{2}}} \biggl(1+\frac{y}{\alpha +\alpha ^{\frac{1}{2}}} \biggr)^{\alpha}e^{-y}\,dy -1 \biggr] \\ &\hphantom{ \quad \Leftrightarrow\quad}\quad < \bigl(\alpha -\alpha ^{\frac{1}{2}}\bigr)^{\alpha}e^{-\alpha +\alpha ^{\frac{1}{2}}} \biggl[ \int _{0}^{1-(\alpha +1)^{\frac{1}{2}}+\alpha ^{\frac{1}{2}}} \biggl(1+\frac{y}{\alpha -\alpha ^{\frac{1}{2}}} \biggr)^{\alpha}e^{-y}\,dy -1 \biggr]. \end{aligned}$$

The remainder of this section is devoted to proving the following inequality:

3.4 $$\begin{aligned}& \int _{0}^{1+(\alpha +1)^{\frac{1}{2}}-\alpha ^{\frac{1}{2}}} \biggl(1+ \frac{y}{\alpha +\alpha ^{\frac{1}{2}}} \biggr)^{\alpha}e^{-y}\,dy \\& \quad < 1< \int _{0}^{1-(\alpha +1)^{\frac{1}{2}}+\alpha ^{\frac{1}{2}}} \biggl(1+ \frac{y}{\alpha -\alpha ^{\frac{1}{2}}} \biggr)^{\alpha}e^{-y}\,dy ,\quad \forall \alpha >1, \end{aligned}$$

which implies (3.1). It is a bit surprising that inequality (3.4) is very delicate, which seems to be unknown in the literature. □

### Proof of the “<1” part of inequality (3.4)

Note that

$$\begin{aligned} &\frac{d}{dy} \biggl[ \biggl(1+\frac{y}{\alpha +\alpha ^{\frac{1}{2}}} \biggr)^{\alpha}e^{-y} \biggr]' \\ &\quad = \frac{\alpha}{\alpha +\alpha ^{\frac{1}{2}}} \biggl(1+ \frac{y}{\alpha +\alpha ^{\frac{1}{2}}} \biggr)^{\alpha -1}e^{-y}- \biggl(1+\frac{y}{\alpha +\alpha ^{\frac{1}{2}}} \biggr)^{\alpha}e^{-y} \\ &\quad = \frac{- (\alpha ^{\frac{1}{2}}+y )}{ \alpha +\alpha ^{\frac{1}{2}}} \biggl(1+ \frac{y}{\alpha +\alpha ^{\frac{1}{2}}} \biggr)^{\alpha -1}e^{-y} \\ &\quad < 0. \end{aligned}$$

Then we have

$$\begin{aligned} &\int _{0}^{1+(\alpha +1)^{\frac{1}{2}}-\alpha ^{\frac{1}{2}}} \biggl(1+ \frac{y}{\alpha +\alpha ^{\frac{1}{2}}} \biggr)^{\alpha}e^{-y}\,dy \\ &\quad < \int _{0}^{1} \biggl(1+\frac{y}{\alpha +\alpha ^{\frac{1}{2}}} \biggr)^{\alpha}e^{-y}\,dy + \bigl[(\alpha +1)^{\frac{1}{2}}- \alpha ^{\frac{1}{2}} \bigr] \biggl(1+\frac{1}{\alpha +\alpha ^{\frac{1}{2}}} \biggr)^{\alpha}e^{-1}. \end{aligned}$$

Hence, to prove the “<1” part of inequality (3.4), it suffices to show that

$$ \bigl[(\alpha +1)^{\frac{1}{2}}-\alpha ^{\frac{1}{2}} \bigr] \biggl(1+ \frac{1}{\alpha +\alpha ^{\frac{1}{2}}} \biggr)^{\alpha}e^{-1}< \int _{0}^{1} \biggl[1- \biggl(1+ \frac{y}{\alpha +\alpha ^{\frac{1}{2}}} \biggr)^{\alpha}e^{-y} \biggr]\,dy . $$

We have

$$\begin{aligned} &\frac{d}{dy} \biggl[1- \biggl(1+ \frac{y}{\alpha +\alpha ^{\frac{1}{2}}} \biggr)^{\alpha}e^{-y} \biggr] \\ &\quad = -\frac{\alpha}{\alpha +\alpha ^{\frac{1}{2}}} \biggl(1+ \frac{y}{\alpha +\alpha ^{\frac{1}{2}}} \biggr)^{\alpha -1}e^{-y} + \biggl(1+\frac{y}{\alpha +\alpha ^{\frac{1}{2}}} \biggr)^{\alpha}e^{-y} \\ &\quad = \frac{\alpha ^{\frac{1}{2}}+y}{\alpha +\alpha ^{\frac{1}{2}}} \biggl(1+\frac{y}{\alpha +\alpha ^{\frac{1}{2}}} \biggr)^{\alpha -1}e^{-y} \\ &\quad > 0 \end{aligned}$$

and

$$\begin{aligned} &\frac{d^{2}}{dy^{2}} \biggl[1- \biggl(1+ \frac{y}{\alpha +\alpha ^{\frac{1}{2}}} \biggr)^{\alpha}e^{-y} \biggr] \\ &\quad = \frac{2\alpha}{\alpha +\alpha ^{\frac{1}{2}}} \biggl(1+ \frac{y}{\alpha +\alpha ^{\frac{1}{2}}} \biggr)^{\alpha -1}e^{-y} - \frac{\alpha (\alpha -1)}{(\alpha +\alpha ^{\frac{1}{2}})^{2}} \biggl(1+ \frac{y}{\alpha +\alpha ^{\frac{1}{2}}} \biggr)^{\alpha -2}e^{-y} \\ &\quad \quad {}- \biggl(1+\frac{y}{\alpha +\alpha ^{\frac{1}{2}}} \biggr)^{\alpha}e^{-y} \\ &\quad = \frac{-y(2\alpha ^{\frac{1}{2}}+y)}{(\alpha +\alpha ^{\frac{1}{2}})^{2}} \biggl(1+\frac{y}{\alpha +\alpha ^{\frac{1}{2}}} \biggr)^{\alpha -2}e^{-y} \\ &\quad < 0. \end{aligned}$$

Then, for fixed *α*, $1- (1+\frac{y}{\alpha +\alpha ^{\frac{1}{2}}} )^{\alpha}e^{-y}$ is an increasing and concave function of *y* on $[0,1]$. Hence, we have

$$\begin{aligned} &\int _{0}^{1} \biggl[1- \biggl(1+ \frac{y}{\alpha +\alpha ^{\frac{1}{2}}} \biggr)^{\alpha}e^{-y} \biggr]\,dy \\ &\quad > \frac{2}{4} \biggl[1- \biggl(1+ \frac{1/2}{\alpha +\alpha ^{\frac{1}{2}}} \biggr)^{\alpha}e^{-1/2} \biggr]+\frac{1}{4} \biggl[1- \biggl(1+ \frac{1}{\alpha +\alpha ^{\frac{1}{2}}} \biggr)^{\alpha}e^{-1} \biggr]. \end{aligned}$$

Thus, to complete the proof of the “<1” part of inequality (3.4), it suffices to show that

$$\begin{aligned} &\bigl[(\alpha +1)^{\frac{1}{2}}-\alpha ^{\frac{1}{2}} \bigr] \biggl(1+ \frac{1}{\alpha +\alpha ^{\frac{1}{2}}} \biggr)^{\alpha}e^{-1} \\ &\quad < \frac{2}{4} \biggl[1- \biggl(1+ \frac{1/2}{\alpha +\alpha ^{\frac{1}{2}}} \biggr)^{\alpha}e^{-1/2} \biggr]+\frac{1}{4} \biggl[1- \biggl(1+ \frac{1}{\alpha +\alpha ^{\frac{1}{2}}} \biggr)^{\alpha}e^{-1} \biggr], \end{aligned}$$

which is equivalent to

3.5 $$\begin{aligned} \bigl\{ 1+4 \bigl[(\alpha +1)^{\frac{1}{2}}-\alpha ^{\frac{1}{2}} \bigr] \bigr\} \biggl(1+\frac{1}{\alpha +\alpha ^{\frac{1}{2}}} \biggr)^{\alpha}e^{-1}+2 \biggl(1+ \frac{1/2}{\alpha +\alpha ^{\frac{1}{2}}} \biggr)^{\alpha}e^{-1/2}< 3, \quad \forall \alpha >1. \end{aligned}$$

The proof of (3.5) will be given in Sect. 4.

### Proof of the “>1” part of inequality (3.4)

Note that

$$ 1-4\bigl[(\alpha +1)^{\frac{1}{2}}-\alpha ^{\frac{1}{2}}\bigr]=0 \quad \Leftrightarrow \quad \alpha = \biggl(\frac{15}{8} \biggr)^{2}. $$

We consider the three cases $1<\alpha \le 2$, $2<\alpha < (\frac{15}{8} )^{2}$, and $\alpha \ge (\frac{15}{8} )^{2}$ separately.

*Case* 1: $1<\alpha \le 2$. By Taylor’s formula, we have

$$\begin{aligned} &\int _{0}^{1-(\alpha +1)^{\frac{1}{2}}+\alpha ^{\frac{1}{2}}} \biggl(1+ \frac{y}{\alpha -\alpha ^{\frac{1}{2}}} \biggr)^{\alpha}e^{-y}\,dy -1 \\ &\quad > \int _{0}^{1-(\alpha +1)^{\frac{1}{2}}+\alpha ^{\frac{1}{2}}} \biggl(1+\frac{\alpha y}{\alpha -\alpha ^{\frac{1}{2}}} \biggr)e^{-y}\,dy -1 \\ &\quad = \frac{\alpha}{\alpha -\alpha ^{\frac{1}{2}}}- \frac{3\alpha +(\alpha -1)\alpha ^{\frac{1}{2}} -\alpha (\alpha +1)^{\frac{1}{2}}}{\alpha -\alpha ^{\frac{1}{2}}} \cdot e^{- [1-(\alpha +1)^{\frac{1}{2}}+\alpha ^{\frac{1}{2}} ]} \\ &\quad = \frac{\alpha e^{- [1-(\alpha +1)^{\frac{1}{2}}+\alpha ^{\frac{1}{2}} ]}}{\alpha -\alpha ^{\frac{1}{2}}} \bigl[e^{1-(\alpha +1)^{\frac{1}{2}}+\alpha ^{\frac{1}{2}}}-3-( \alpha -1)\alpha ^{-\frac{1}{2}}+(\alpha +1)^{\frac{1}{2}} \bigr] \\ &\quad = \frac{\alpha e^{- [1-(\alpha +1)^{\frac{1}{2}}+\alpha ^{\frac{1}{2}} ]}}{\alpha -\alpha ^{\frac{1}{2}}} \bigl\{ e^{1-(\alpha +1)^{\frac{1}{2}}+\alpha ^{\frac{1}{2}}} +\bigl[( \alpha +1)^{\frac{1}{2}}-\alpha ^{\frac{1}{2}}\bigr]-3+\alpha ^{-\frac{1}{2}} \bigr\} . \end{aligned}$$

Set

$$ w:=(\alpha +1)^{\frac{1}{2}}-\alpha ^{\frac{1}{2}}. $$

Then

$$ \xi :=\frac{1}{2^{\frac{1}{2}}+3^{\frac{1}{2}}}\le w< \frac{1}{1+2^{\frac{1}{2}}} $$

and

$$ \alpha ^{-\frac{1}{2}}=\frac{2w}{1-w^{2}}. $$

We have

$$\begin{aligned} e^{1-(\alpha +1)^{\frac{1}{2}}+\alpha ^{\frac{1}{2}}} +\bigl[(\alpha +1)^{\frac{1}{2}}- \alpha ^{\frac{1}{2}} \bigr]-3+\alpha ^{-\frac{1}{2}} =e^{1-w}+w-3+ \frac{2w}{1-w^{2}} \end{aligned}$$

and

$$\begin{aligned} \biggl(e^{1-w}+w-3+\frac{2w}{1-w^{2}} \biggr)' & = -e^{1-w}+1+ \frac{2(1+w^{2})}{(1-w^{2})^{2}} \\ & \ge -e^{1-\xi}+1+\frac{2(1+\xi ^{2})}{(1-\xi ^{2})^{2}} \\ & = 1.746594. \end{aligned}$$

Then

$$\begin{aligned} &e^{1-(\alpha +1)^{\frac{1}{2}}+\alpha ^{\frac{1}{2}}} +\bigl[(\alpha +1)^{\frac{1}{2}}- \alpha ^{\frac{1}{2}}\bigr]-3+\alpha ^{-\frac{1}{2}} \\ &\quad \ge e^{1-\xi}+\xi -3+\frac{2\xi}{1-\xi ^{2}} \\ &\quad = 0.003095392. \end{aligned}$$

Thus, the “>1” part of inequality (3.4) holds for $1<\alpha \le 2$.

*Case* 2: $2<\alpha < (\frac{15}{8} )^{2}$. By Taylor’s formula, we have

$$\begin{aligned} &\int _{0}^{1-(\alpha +1)^{\frac{1}{2}}+\alpha ^{\frac{1}{2}}} \biggl(1+ \frac{y}{\alpha -\alpha ^{\frac{1}{2}}} \biggr)^{\alpha}e^{-y}\,dy -1 \\ &\quad > \int _{0}^{1-(\alpha +1)^{\frac{1}{2}}+\alpha ^{\frac{1}{2}}} \biggl[1+\frac{\alpha y}{\alpha -\alpha ^{\frac{1}{2}}}+ \frac{\alpha (\alpha -1) y^{2}}{2(\alpha -\alpha ^{\frac{1}{2}})^{2}} \biggr]e^{-y}\,dy -1 \\ &\quad = \frac{2\alpha ^{\frac{1}{2}}+1}{\alpha ^{\frac{1}{2}}-1}- \frac{\alpha \alpha ^{\frac{1}{2}}-\alpha (\alpha +1)^{\frac{1}{2}}+4\alpha -4[\alpha (\alpha +1)]^{\frac{1}{2}}+8\alpha ^{\frac{1}{2}}-2(\alpha +1)^{\frac{1}{2}}+2}{\alpha ^{\frac{1}{2}}-1} \\ &\quad \quad {}\cdot e^{- [1-(\alpha +1)^{\frac{1}{2}}+\alpha ^{\frac{1}{2}} ]} \\ &\quad = \frac{e^{- [1-(\alpha +1)^{\frac{1}{2}}+\alpha ^{\frac{1}{2}} ]}}{ \alpha ^{\frac{1}{2}-1}} \\ &\quad \quad{} \cdot \bigl\{ \bigl(2\alpha ^{\frac{1}{2}}+1\bigr)e^{1-(\alpha +1)^{\frac{1}{2}}+\alpha ^{\frac{1}{2}}}- \bigl[ \alpha \alpha ^{\frac{1}{2}}-\alpha (\alpha +1)^{\frac{1}{2}}+4 \alpha - 4 \bigl[\alpha (\alpha +1)\bigr]^{\frac{1}{2}} \\ &\quad \quad {}+8\alpha ^{\frac{1}{2}}-2( \alpha +1)^{\frac{1}{2}}+2 \bigr] \bigr\} \\ &\quad > \frac{e^{-[1-(\alpha +1)^{\frac{1}{2}}+\alpha ^{\frac{1}{2}}]}}{ \alpha ^{\frac{1}{2}-1}} \Biggl\{ \bigl(2\alpha ^{\frac{1}{2}}+1\bigr) \sum _{n=0}^{4} \frac{ [1-(\alpha +1)^{\frac{1}{2}}+\alpha ^{\frac{1}{2}} ]^{n}}{n!} \\ &\quad \quad - \bigl[\alpha \alpha ^{\frac{1}{2}}-\alpha ( \alpha +1)^{\frac{1}{2}}+4\alpha - 4\bigl[\alpha (\alpha +1)\bigr]^{\frac{1}{2}}+8 \alpha ^{\frac{1}{2}}-2(\alpha +1)^{\frac{1}{2}}+2 \bigr] \Biggr\} . \end{aligned}$$

Define

$$\begin{aligned} J&: = \bigl(2\alpha ^{\frac{1}{2}}+1\bigr)\sum_{n=0}^{4} \frac{ [1-(\alpha +1)^{\frac{1}{2}}+\alpha ^{\frac{1}{2}} ]^{n}}{n!} \\ &\quad{} - \bigl[\alpha \alpha ^{\frac{1}{2}}-\alpha (\alpha +1)^{\frac{1}{2}}+4 \alpha - 4\bigl[\alpha (\alpha +1)\bigr]^{\frac{1}{2}}+8\alpha ^{\frac{1}{2}}-2( \alpha +1)^{\frac{1}{2}}+2 \bigr]. \end{aligned}$$

Set

$$ w:=(\alpha +1)^{\frac{1}{2}}-\alpha ^{\frac{1}{2}}. $$

Then

$$ \frac{1}{4}< w< \frac{1}{2^{\frac{1}{2}}+3^{\frac{1}{2}}},\qquad \alpha ^{\frac{1}{2}}= \frac{1-w^{2}}{2w},\qquad (\alpha +1)^{\frac{1}{2}}= \frac{1+w^{2}}{2w}. $$

Hence,

$$\begin{aligned} J& = \frac{1+w-w^{2}}{w}\sum_{n=0}^{4} \frac{(1-w)^{n}}{n!}+ \frac{w^{4}-8w^{3}+18w^{2}-11}{4w} \\ & = \frac{-1 + w + 9 w^{2} + 38 w^{3} - 31 w^{4} + 9 w^{5} - w^{6}}{24 w} \\ & > \frac{-1 + \frac{1}{4} + (9) (\frac{1}{4} )^{2} + (7)(\frac{1}{4})^{3}+ (31) (\frac{1}{4})^{3}(\frac{1}{2}) +(8)(\frac{1}{4})^{5} +(\frac{1}{4})^{5}(\frac{1}{2})}{24 w} \\ & = \frac{0.1723633}{24w} \\ & > 0. \end{aligned}$$

Thus, the “>1” part of inequality (3.4) holds for $2<\alpha < (\frac{15}{8} )^{2}$.

*Case* 3: $\alpha \ge (\frac{15}{8} )^{2}$. Note that for $0\leq y\leq 1-(\alpha +1)^{\frac{1}{2}}+\alpha ^{\frac{1}{2}}$, we have

$$\begin{aligned} &\frac{d}{dy} \biggl[ \biggl(1+\frac{y}{\alpha -\alpha ^{\frac{1}{2}}} \biggr)^{\alpha}e^{-y} \biggr]' \\ &\quad = \frac{\alpha}{\alpha -\alpha ^{\frac{1}{2}}} \biggl(1+ \frac{y}{\alpha -\alpha ^{\frac{1}{2}}} \biggr)^{\alpha -1}e^{-y}- \biggl(1+\frac{y}{\alpha -\alpha ^{\frac{1}{2}}} \biggr)^{\alpha}e^{-y} \\ &\quad = \frac{\alpha ^{\frac{1}{2}}-y}{\alpha -\alpha ^{\frac{1}{2}}} \biggl(1+\frac{y}{\alpha -\alpha ^{\frac{1}{2}}} \biggr)^{\alpha -1}e^{-y} \\ &\quad > 0. \end{aligned}$$

Then

$$\begin{aligned} &\int _{0}^{1-(\alpha +1)^{\frac{1}{2}}+\alpha ^{\frac{1}{2}}} \biggl(1+ \frac{y}{\alpha -\alpha ^{\frac{1}{2}}} \biggr)^{\alpha}e^{-y}\,dy \\ &\quad > \int _{0}^{1} \biggl(1+\frac{y}{\alpha -\alpha ^{\frac{1}{2}}} \biggr)^{\alpha}e^{-y}\,dy - \bigl[(\alpha +1)^{\frac{1}{2}}- \alpha ^{\frac{1}{2}} \bigr] \biggl(1+\frac{1}{\alpha -\alpha ^{\frac{1}{2}}} \biggr)^{\alpha}e^{-1}. \end{aligned}$$

Hence, to prove the “>1” part of inequality (3.4), it suffices to show that

$$ \bigl[(\alpha +1)^{\frac{1}{2}}-\alpha ^{\frac{1}{2}} \bigr] \biggl(1+ \frac{1}{\alpha -\alpha ^{\frac{1}{2}}} \biggr)^{\alpha}e^{-1}< \int _{0}^{1} \biggl[ \biggl(1+ \frac{y}{\alpha -\alpha ^{\frac{1}{2}}} \biggr)^{\alpha}e^{-y}-1 \biggr]\,dy . $$

For $y\in [0,1]$, we have

$$\begin{aligned} &\frac{d}{dy} \biggl[ \biggl(1+\frac{y}{\alpha -\alpha ^{\frac{1}{2}}} \biggr)^{\alpha}e^{-y}-1 \biggr] \\ &\quad = \frac{\alpha}{\alpha -\alpha ^{\frac{1}{2}}} \biggl(1+ \frac{y}{\alpha -\alpha ^{\frac{1}{2}}} \biggr)^{\alpha -1}e^{-y}- \biggl(1+\frac{y}{\alpha -\alpha ^{\frac{1}{2}}} \biggr)^{\alpha}e^{-y} \\ &\quad = \frac{\alpha ^{\frac{1}{2}}-y}{\alpha -\alpha ^{\frac{1}{2}}} \biggl(1+\frac{y}{\alpha -\alpha ^{\frac{1}{2}}} \biggr)^{\alpha -1}e^{-y} \\ &\quad > 0 \end{aligned}$$

and

$$\begin{aligned} &\frac{d^{2}}{dy^{2}} \biggl[ \biggl(1+ \frac{y}{\alpha -\alpha ^{\frac{1}{2}}} \biggr)^{\alpha}e^{-y}-1 \biggr] \\ &\quad = -\frac{2\alpha}{\alpha -\alpha ^{\frac{1}{2}}} \biggl(1+ \frac{y}{\alpha -\alpha ^{\frac{1}{2}}} \biggr)^{\alpha -1}e^{-y} + \frac{\alpha (\alpha -1)}{(\alpha -\alpha ^{\frac{1}{2}})^{2}} \biggl(1+ \frac{y}{\alpha -\alpha ^{\frac{1}{2}}} \biggr)^{\alpha -2}e^{-y} \\ &\quad \quad {}+ \biggl(1+\frac{y}{\alpha -\alpha ^{\frac{1}{2}}} \biggr)^{\alpha}e^{-y} \\ &\quad = \frac{-y(2\alpha ^{\frac{1}{2}}-y)}{(\alpha -\alpha ^{\frac{1}{2}})^{2}} \biggl(1+\frac{y}{\alpha -\alpha ^{\frac{1}{2}}} \biggr)^{\alpha -2}e^{-y} \\ &\quad < 0. \end{aligned}$$

Then, for fixed *α*, $(1+\frac{y}{\alpha -\alpha ^{\frac{1}{2}}} )^{\alpha}e^{-y}-1$ is an increasing and concave function of *y* on $[0,1]$. Hence, we have

$$\begin{aligned} &\int _{0}^{1} \biggl[ \biggl(1+ \frac{y}{\alpha -\alpha ^{\frac{1}{2}}} \biggr)^{\alpha}e^{-y}-1 \biggr]\,dy \\ &\quad > \frac{2}{4} \biggl[ \biggl(1+ \frac{1/2}{\alpha -\alpha ^{\frac{1}{2}}} \biggr)^{\alpha}e^{-1/2}-1 \biggr]+\frac{1}{4} \biggl[ \biggl(1+ \frac{1}{\alpha -\alpha ^{\frac{1}{2}}} \biggr)^{\alpha}e^{-1}-1 \biggr]. \end{aligned}$$

Thus, to complete the proof of the “>1” part of inequality (3.4), it suffices to show that

$$\begin{aligned} &\bigl[(\alpha +1)^{\frac{1}{2}}-\alpha ^{\frac{1}{2}} \bigr] \biggl(1+ \frac{1}{\alpha -\alpha ^{\frac{1}{2}}} \biggr)^{\alpha}e^{-1} \\ &\quad < \frac{2}{4} \biggl[ \biggl(1+ \frac{1/2}{\alpha -\alpha ^{\frac{1}{2}}} \biggr)^{\alpha}e^{-1/2}-1 \biggr]+\frac{1}{4} \biggl[ \biggl(1+ \frac{1}{\alpha -\alpha ^{\frac{1}{2}}} \biggr)^{\alpha}e^{-1}-1 \biggr], \end{aligned}$$

which is equivalent to

3.6 $$\begin{aligned}& \bigl\{ 1-4\bigl[(\alpha +1)^{\frac{1}{2}}-\alpha ^{\frac{1}{2}}\bigr] \bigr\} \biggl(1+\frac{1}{\alpha -\alpha ^{\frac{1}{2}}} \biggr)^{\alpha}e^{-1}+2 \biggl(1+\frac{1/2}{\alpha -\alpha ^{\frac{1}{2}}} \biggr)^{\alpha}e^{-1/2} \\& \quad >3, \quad \forall \alpha \ge \biggl( \frac{15}{8} \biggr)^{2}. \end{aligned}$$

The proof of (3.6) will be given in Sect. 4.

## Proofs of inequalities (3.5) and (3.6)

### Proof of (3.5)

Define

$$ \tau _{+}:=\frac{1}{\alpha +\alpha ^{\frac{1}{2}}},\qquad \eta _{+}:= \frac{\tau _{+}}{2}. $$

We have

4.1 $$\begin{aligned} &\bigl\{ 1+4\bigl[(\alpha +1)^{\frac{1}{2}}-\alpha ^{\frac{1}{2}}\bigr] \bigr\} \biggl(1+\frac{1}{\alpha +\alpha ^{\frac{1}{2}}} \biggr)^{\alpha}e^{-1}+ 2 \biggl(1+\frac{1/2}{\alpha +\alpha ^{\frac{1}{2}}} \biggr)^{\alpha}e^{-1/2} \\ &\quad = \bigl\{ 1+4\bigl[(\alpha +1)^{\frac{1}{2}}-\alpha ^{\frac{1}{2}}\bigr] \bigr\} e^{-1+\alpha \ln (1+\tau _{+})}+2e^{-\frac{1}{2}+\alpha \ln (1+\eta _{+})} \\ &\quad < \bigl\{ 1+4\bigl[(\alpha +1)^{\frac{1}{2}}-\alpha ^{\frac{1}{2}}\bigr] \bigr\} e^{-1+\alpha (\tau _{+}-\frac{\tau _{+}^{2}}{2}+\frac{\tau _{+}^{3}}{3}-\frac{\tau _{+}^{4}}{4} +\frac{\tau _{+}^{5}}{5} )}+2e^{-\frac{1}{2}+\alpha (\eta _{+}-\frac{\eta _{+}^{2}}{2}+ \frac{\eta _{+}^{3}}{3}-\frac{\eta _{+}^{4}}{4}+\frac{\eta _{+}^{5}}{5} )}. \end{aligned}$$

Set

$$ w:=(\alpha +1)^{\frac{1}{2}}-\alpha ^{\frac{1}{2}}. $$

Then, by the condition $\alpha >1$, we get

$$ 0< w< \frac{1}{2},\qquad 1-w^{2}>0,\qquad 1+2w-w^{2}>0 $$

and

$$ \alpha =\frac{(1-w^{2})^{2}}{4w^{2}},\qquad \tau _{+}= \frac{4w^{2}}{(1-w^{2})(1+2w-w^{2})},\qquad \eta _{+}= \frac{2w^{2}}{(1-w^{2})(1+2w-w^{2})}. $$

Define

$$ P_{+}:=-1+\alpha \biggl(\tau _{+}-\frac{\tau _{+}^{2}}{2}+\frac{\tau _{+}^{3}}{3}- \frac{\tau _{+}^{4}}{4}+\frac{\tau _{+}^{5}}{5} \biggr),\qquad Q_{+}:=- \frac{1}{2}+\alpha \biggl(\eta _{+}-\frac{\eta _{+}^{2}}{2}+ \frac{\eta _{+}^{3}}{3}-\frac{\eta _{+}^{4}}{4}+\frac{\eta _{+}^{5}}{5} \biggr). $$

By virtue of *Mathematica*, we get

$$\begin{aligned} F_{+}&: = 15\bigl(1 - w^{2}\bigr)^{3} \bigl(1+2 w - w^{2}\bigr)^{5}P_{+} \\ & = 2 w \bigl(-15 - 135 w - 345 w^{2} + 190 w^{3} + 1735 w^{4} + 495 w^{5} - 3615 w^{6}- 716 w^{7} \\ &\quad + 3615 w^{8}+ 495 w^{9} - 1735 w^{10} + 190 w^{11} + 345 w^{12} - 135 w^{13} + 15 w^{14}\bigr). \end{aligned}$$

Set

4.2 $$\begin{aligned} w:=\frac{1}{2(1+q^{2})}. \end{aligned}$$

We have

$$\begin{aligned} G_{+}&: = 16{,}384 \bigl(1 + q^{2}\bigr)^{14}\cdot \frac{F_{+}}{2w} \\ & = -1{,}140{,}603 - 17{,}129{,}046 q^{2} - 115{,}786{,}348 q^{4} - 468{,}301{,}840 q^{6} \\ &\quad {}- 1{,}267{,}262{,}160 q^{8} - 2{,}427{,}446{,}688 q^{10} - 3{,}393{,}664{,}576 q^{12} - 3{,}517{,}163{,}008 q^{14} \\ &\quad {} - 2{,}715{,}321{,}600 q^{16} - 1{,}554{,}209{,}280 q^{18} - 649{,}507{,}840 q^{20} \\ &\quad{} - 192{,}286{,}720 q^{22} - 38{,}154{,}240 q^{24} - 4{,}546{,}560 q^{26} - 245{,}760 q^{28}, \end{aligned}$$

which implies that $P_{+}$ is negative. By virtue of *Mathematica*, we get

$$\begin{aligned} H_{+}&: = 30 \bigl(1 - w^{2}\bigr)^{3} \bigl(1 + 2 w - w^{2}\bigr)^{5}Q_{+} \\ & = w \bigl(-30 - 255 w - 600 w^{2} + 410 w^{3} + 2900 w^{4} + 705 w^{5} - 5550 w^{6} - 1672 w^{7} \\ &\quad + 5550 w^{8}+ 705 w^{9} - 2900 w^{10}+ 410 w^{11} + 600 w^{12} - 255 w^{13} + 30 w^{14}\bigr). \end{aligned}$$

Further, by the transformation (4.2), we obtain

$$\begin{aligned} I_{+}&: = 8192 \bigl(1 + q^{2}\bigr)^{14}\cdot \frac{H_{+}}{w} \\ & = -1{,}083{,}048 - 16{,}069{,}911 q^{2} - 108{,}024{,}568 q^{4} - 435{,}858{,}040 q^{6} - 1{,}178{,}745{,}360 q^{8} \\ &\quad{} - 2{,}259{,}543{,}408 q^{10}- 3{,}165{,}284{,}416 q^{12} - 3{,}291{,}555{,}328 q^{14} - 2{,}553{,}515{,}520 q^{16} \\ &\quad {}- 1{,}471{,}031{,}040 q^{18} - 619{,}724{,}800 q^{20}- 185{,}251{,}840 q^{22} \\ &\quad{} - 37{,}171{,}200 q^{24} - 4{,}485{,}120 q^{26} - 245{,}760 q^{28}, \end{aligned}$$

which implies that $Q_{+}$ is negative. Thus, by (4.1), we get

4.3 $$\begin{aligned} &\bigl\{ 1+4\bigl[(\alpha +1)^{\frac{1}{2}}-\alpha ^{\frac{1}{2}}\bigr] \bigr\} \biggl(1+\frac{1}{\alpha +\alpha ^{\frac{1}{2}}} \biggr)^{\alpha}e^{-1}+2 \biggl(1+\frac{1/2}{\alpha +\alpha ^{\frac{1}{2}}} \biggr)^{\alpha}e^{-1/2}-3 \\ &\quad < (1+4w)e^{P_{+}}+2e^{Q_{+}}-3 \\ &\quad < (1+4w) \biggl(1+P_{+}+\frac{P_{+}^{2}}{2}+\frac{P_{+}^{3}}{3!}+ \frac{P_{+}^{4}}{4!} \biggr)+2 \biggl(1+Q_{+}+\frac{Q_{+}^{2}}{2}+ \frac{Q_{+}^{3}}{3!}+\frac{Q_{+}^{4}}{4!} \biggr)-3 \\ &\quad : = R_{+}. \end{aligned}$$

Define

$$ L_{+}:=9{,}720{,}000 \bigl(1 - w^{2}\bigr)^{12} \bigl(1 + 2 w - w^{2}\bigr)^{20}w^{-3}R_{+} $$

and

$$ V_{+}:=-18{,}014{,}398{,}509{,}481{,}984 \bigl(1 + q^{2}\bigr)^{62}L_{+}. $$

By virtue of *Mathematica*, we obtain the expansion of the polynomial $V_{+}(q)$ (see Sect. 5). Note that all terms in the expansion are positive. Then $R_{+}$ is negative. Therefore, the proof is complete by (4.3).

### Proof of (3.6)

Define

$$ \tau _{-}:=\frac{1}{\alpha -\alpha ^{\frac{1}{2}}},\qquad \eta _{-}:= \frac{\tau _{-}}{2}. $$

We have

4.4 $$\begin{aligned} &\bigl\{ 1-4\bigl[(\alpha +1)^{\frac{1}{2}}-\alpha ^{\frac{1}{2}}\bigr] \bigr\} \biggl(1+\frac{1}{\alpha -\alpha ^{\frac{1}{2}}} \biggr)^{\alpha}e^{-1}+ 2 \biggl(1+\frac{1/2}{\alpha -\alpha ^{\frac{1}{2}}} \biggr)^{\alpha}e^{-1/2} \\ &\quad = \bigl\{ 1-4\bigl[(\alpha +1)^{\frac{1}{2}}-\alpha ^{\frac{1}{2}}\bigr] \bigr\} e^{-1+\alpha \ln (1+\tau _{-})}+2e^{-\frac{1}{2}+\alpha \ln (1+\eta _{-})} \\ &\quad > \bigl\{ 1-4\bigl[(\alpha +1)^{\frac{1}{2}}-\alpha ^{\frac{1}{2}}\bigr] \bigr\} e^{-1+\alpha (\tau _{-}-\frac{\tau _{-}^{2}}{2}+\frac{\tau _{-}^{3}}{3}-\frac{\tau _{-}^{4}}{4} )} +2e^{-\frac{1}{2}+\alpha (\eta _{-}-\frac{\eta _{-}^{2}}{2}+\frac{\eta _{-}^{3}}{3}- \frac{\eta _{-}^{4}}{4} )}. \end{aligned}$$

Set

$$ w:=(\alpha +1)^{\frac{1}{2}}-\alpha ^{\frac{1}{2}}. $$

Then, by the condition $\alpha \geq (\frac{15}{8} )^{2}$, we have

$$ 0< w\le \frac{1}{4},\qquad 1-w^{2}>0,\qquad 1-2w-w^{2}>0 $$

and

$$ \alpha =\frac{(1-w^{2})^{2}}{4w^{2}},\qquad \tau _{-}= \frac{4w^{2}}{(1-w^{2})(1-2w-w^{2})},\qquad \eta _{-}= \frac{2w^{2}}{(1-w^{2})(1-2w-w^{2})}. $$

Define

$$ P_{-}:=-1+\alpha \biggl(\tau _{-}-\frac{\tau _{-}^{2}}{2}+\frac{\tau _{-}^{3}}{3}- \frac{\tau _{-}^{4}}{4} \biggr),\qquad Q_{-}:=-\frac{1}{2}+\alpha \biggl( \eta _{-}-\frac{\eta _{-}^{2}}{2}+\frac{\eta _{-}^{3}}{3}-\frac{\eta _{-}^{4}}{4} \biggr). $$

We have

$$\begin{aligned} F_{-}&: = 3 \bigl(1 - w^{2}\bigr)^{2} \bigl(1 - 2 w - w^{2}\bigr)^{4}P_{-} \\ & = -2 w \bigl(-3 + 21 w - 33 w^{2} - 56 w^{3} + 130 w^{4} + 94 w^{5} \\ &\quad {}- 130 w^{6} - 56 w^{7} +33 w^{8} + 21 w^{9} + 3 w^{10}\bigr). \end{aligned}$$

Set

4.5 $$\begin{aligned} w:=\frac{1}{4(1+q^{2})}. \end{aligned}$$

We get

$$\begin{aligned} G_{-}&: = 1{,}048{,}576 \bigl(1 + q^{2}\bigr)^{10}\cdot \frac{F_{-}}{2w} \\ & = 128{,}409 + 2{,}102{,}668 q^{2} + 14{,}459{,}888 q^{4} + 56{,}813{,}056 q^{6} \\ &\quad {}+ 142{,}035{,}456 q^{8} + 236{,}177{,}408 q^{10} + 264{,}626{,}176 q^{12} \\ &\quad{}+ 197{,}525{,}504 q^{14} + 94{,}175{,}232 q^{16} + 25{,}952{,}256 q^{18} + 3{,}145{,}728 q^{20}, \end{aligned}$$

which implies that $P_{-}$ is positive. We have

$$\begin{aligned} H_{-}&: = 6 \bigl(1 - w^{2}\bigr)^{2} \bigl(1 - 2 w - w^{2}\bigr)^{4}Q_{-} \\ & = w \bigl(6 - 39 w + 54 w^{2} + 100 w^{3} - 200 w^{4} - 128 w^{5} + 200 w^{6} \\ &\quad {}+ 100 w^{7} - 54 w^{8} - 39 w^{9} - 6 w^{10}\bigr). \end{aligned}$$

By the transformation (4.5), we get

$$\begin{aligned} I_{-}&: = 524{,}288 \bigl(1 + q^{2}\bigr)^{10}\cdot \frac{H_{-}}{w} \\ & = 175{,}743 + 2{,}666{,}962 q^{2} + 17{,}644{,}496 q^{4} + 67{,}116{,}160 q^{6} \\ &\quad{} + 162{,}604{,}032 q^{8}+ 262{,}406{,}144 q^{10}+ 286{,}081{,}024 q^{12} \\ &\quad {}+ 208{,}437{,}248 q^{14} + 97{,}320{,}960 q^{16} + 26{,}345{,}472 q^{18} + 3{,}145{,}728 q^{20}, \end{aligned}$$

which implies that $Q_{-}$ is positive. Thus, by (4.4), we get

4.6 $$\begin{aligned} &\bigl\{ 1-4\bigl[(\alpha +1)^{\frac{1}{2}}-\alpha ^{\frac{1}{2}}\bigr] \bigr\} \biggl(1+\frac{1}{\alpha -\alpha ^{\frac{1}{2}}} \biggr)^{\alpha}e^{-1}+ 2 \biggl(1+\frac{1/2}{\alpha -\alpha ^{\frac{1}{2}}} \biggr)^{\alpha}e^{-1/2}-3 \\ &\quad > (1-4w)e^{P_{-}}+2e^{Q_{-}}-3 \\ &\quad > (1-4w) \biggl(1+P_{-}+\frac{P_{-}^{2}}{2}+\frac{P_{-}^{3}}{3!} \biggr)+2 \biggl(1+Q_{-}+\frac{Q_{-}^{2}}{2}+\frac{Q_{-}^{3}}{3!} \biggr)-3 \\ &\quad : = R_{-}. \end{aligned}$$

Define

$$ L_{-}:=-648 \bigl(1 - w^{2}\bigr)^{6} \bigl(1 - 2 w - w^{2}\bigr)^{12}w^{-3}R_{-} $$

and

$$ V_{-}:=-9{,}223{,}372{,}036{,}854{,}775{,}808 \bigl(1 + q^{2}\bigr)^{34}L_{-}. $$

By virtue of *Mathematica*, we obtain the expansion of the polynomial $V_{-}(q)$ (see Sect. 5). Note that all terms in the expansion are positive. Then $R_{-}$ is positive. Therefore, the proof is complete by (4.6).

## Concluding remarks

It is worth pointing out that the software *Mathematica* plays an important role in our work, which provides us with deep insight into how to handle delicate inequalities. Although it is possible to figure out clever, traditional methods to establish the positiveness of polynomials $V_{+}(q)$ and $V_{-}(q)$ in Sect. 4, it seems more natural to use the computer to quickly present expansions so as to solve the problem. Actually, the computer-assisted proof given for inequality (1.3) also explains why it was not discovered before. We would like to emphasize that all proofs contained in this paper remain rigorous and easily verifiable.

A distribution on $\mathbb{R}$ is infinitely divisible if it can be expressed as the distribution of the sum of an arbitrary number of i.i.d. random variables. We know that the Gamma distribution is infinitely divisible and each Lévy process can be associated with an infinitely divisible distribution (cf. [10, 11]). Motivated by Theorem 1.2, it is natural to ask if any infinitely divisible random variable *L* satisfies the following inequality:

5.1 $$ P \bigl\{ \bigl\vert L-E[L] \bigr\vert \le \sqrt{ \operatorname{Var}(L)} \bigr\} \ge P\bigl\{ \vert Z \vert \le 1\bigr\} . $$

In the forthcoming papers [13, 14], we prove that inequality (5.1) holds for many familiar infinitely divisible continuous distributions including the Laplace, Gumbel, logistic, Pareto, infinitely divisible Weibull, log-normal, Student’s *t*, inverse Gaussian, and *F* distributions.

Inequality (5.1) has the potential to be used in fitting probability distributions to data. Given a data set, we compute the proportion of values that fall within one sample standard deviation of the sample mean. If this proportion is bigger than 0.6827, then we consider using a Gamma distribution or another infinitely divisible distribution that was mentioned above to fit the data. Otherwise, if the proportion is much smaller than 0.6827, then we should avoid using those familiar distributions to describe the data.

## Data Availability

This manuscript has no associated data.

## Appendix: *Mathematica* calculations

The expansion of the polynomial $V_{+}(q)$:
V+=23565171557938261664962395 + 1985238765536369188253388462 q^2 + 76017937191609745093093565184 q^4 + 1815476155917282265018752272232 q^6 + 30868042081839055982554050213660 q^8 + 401897536051918258546845673711320 q^10 + 4195397709111549929883773768957292 q^12 + 36238699732610615067411794056699104 q^14 + 265002286089679374723172860122766982 q^16 + 1669237124849349342077586449716389470 q^18 + 9179934813394932229977676436328785920 q^20 + 44555295354320392501114611345123622400 q^22 + 192537160208281140648975165919934835200 q^24 + 746181252269526741637909507751082171520 q^26 + 2609372572626683435719917787491018652160 q^28 + 8276209631283583168755734561689661224960 q^30 + 23913569456882144063241575623509484876800 q^32 + 63185851825755484161960668172292699909120 q^34 + 153171842040744452342444666253152790732800 q^36 + 341628452529444844018632398179833131991040 q^38 + 702775058219816773204544225960017412751360 q^40 + 1336281286191807830241756821296507838955520 q^42 + 2352931028757956298911312671496544634142720 q^44 + 3842851078067257537573706091171559356497920 q^46 + 5829597689354288278514821031866786159656960 q^48 + 8224048629268397888867021111129844469596160 q^50 + 10800344227796355322915422778734559920914432 q^52 + 13214925874596962589048294078754839901241344 q^54 + 15075437985869745487585690312752934894436352 q^56 + 16043253396277674458218536937392302561689600 q^58 + 15933483495160554733717977505944446676500480 q^60 + 14772181225346687498328707734409833005187072 q^62 + 12786642631638024827680853914736629691449344 q^64 + 10333607587858511627607426462208954269171712 q^66 + 7796135691442288431829216828566360534548480 q^68 + 5489455045169343972022956242977322445045760 q^70 + 3606053976460179205452292516224406577479680 q^72 + 2208802483012122361676530694278736018145280 q^74 + 1260683716848402925787749700503070572544000 q^76 + 669904634456217504368236284579198848204800 q^78 + 331081492020125941589702200450146757509120 q^80 + 152001230583526972614803279225239581491200 q^82 + 64734238129983061042604032362158017740800 q^84 + 25531661312772564369272230408940525977600 q^86 + 9307900274880934185285303838052660019200 q^88 + 3129622227458365558314112917099983667200 q^90 + 968033669852811173795309928049750835200 q^92 + 274640462267821497605095290057418342400 q^94 + 71224038857339584104332613373237657600 q^96 + 16816854347488682979216179348688076800 q^98 + 3598246400042953802386878316412928000 q^100 + 693860503839280523254707460767744000 q^102 + 119794916777653504670468143054848000 q^104 + 18371891299642536871251828277248000 q^106 + 2478647665316721166509051740160000 q^108 + 290656583441325139861690122240000 q^110 + 29171448597603811259616067584000 q^112 + 2455470675466333890778497024000 q^114 + 168582129968383522599075840000 q^116 + 9065459012974557989437440000 q^118 + 358068461185282678456320000 q^120 + 9236522547766697656320000 q^122 + 116733302341443256320000 q^124.

The expansion of the polynomial $V_{-}(q)$:
V-=1058023271132626023 + 51541890229923566472 q^2 + 1213009372688989850064 q^4 + 18352820646596071930240 q^6 + 200442482186879766344000 q^8 + 1682464063207304317242816 q^10 + 11285809233594557704985856 q^12 + 62123650712872430361438720 q^14 + 286006349074965960756670464 q^16 + 1116988330180696358380290048 q^18 + 3741070988530167056939876352 q^20 + 10836728622922107883411734528 q^22 + 27330768999389436608140804096 q^24 + 60330629581678789398471114752 q^26 + 117040259220868123540341129216 q^28 + 200169684615441568277429485568 q^30 + 302487634652646366318853881856 q^32 + 404485176548048584076188188672 q^34 + 478958931700928292722105647104 q^36 + 502214198209105947113507782656 q^38 + 465950173145939141611449483264 q^40 + 381911302204202972478305206272 q^42 + 275855094787796630236532047872 q^44 + 174972859491619945161380855808 q^46 + 97001064005371803174963249152 q^48 + 46708058892337905269349548032 q^50 + 19376798019028218231165812736 q^52 + 6852048396846611541188935680 q^54 + 2036474622748306983572471808 q^56 + 499070059195604547617685504 q^58 + 98184545971566239935365120 q^60 + 14905936080354118622773248 q^62 + 1639091209276985243074560 q^64 + 116172982490204328689664 q^66 + 3984496719921263149056 q^68.
